# 

**Published:** 1848-12

**Authors:** 


*New Publications.*—Several new medical publications have lately been received, which we shall notice, or review, so soon as we have been able to bestow upon them proper examination.

